# Phosphatidylinositol 3‐Kinase δ Deficiency Protects From Antimyeloperoxidase Vasculitis

**DOI:** 10.1002/art.42298

**Published:** 2022-11-18

**Authors:** Fernanda Flórez‐Barrós, Simon Freeley, El Li Tham, Michael G. Robson

**Affiliations:** ^1^ School of Immunology and Microbial Sciences, King's College London, Guy's Hospital London UK

## Abstract

**Objective:**

Antineutrophil cytoplasmic antibody–associated vasculitis (AAV) is a systemic autoimmune disease in which glomerulonephritis is an important manifestation. Antibodies against myeloperoxidase (MPO) or proteinase 3 are thought to be important in pathogenesis. Phosphoinositide 3‐kinase δ (PI3Kδ) mediates a number of effects in lymphocytes, but its role in myeloid cell responses is less clear. Therefore, this study was undertaken to assess this in a preclinical model of glomerulonephritis induced by the transfer of antibodies to MPO.

**Methods:**

D910A mice with inactive PI3Kδ were compared with wild‐type controls. Disease protocols allowed for a comparison of experimental groups in the setting of both mild and more severe disease. Adoptive transfer experiments were performed, with flow cytometric analysis of digested kidneys taken at the end of the experiment.

**Results:**

With mild disease, D910A mice had fewer glomerular macrophages, fewer glomerular neutrophils, and reduced albuminuria compared with wild‐type controls. With more severe disease, they also had fewer glomerular crescents and lower serum creatinine levels, indicating protection from acute kidney injury. Adoptive transfer experiments showed a defect in the recruitment of D910A monocytes to the diseased kidney.

**Conclusion:**

Mice with inactive PI3Kδ were protected from anti‐MPO vasculitis. This is due to cell intrinsic defect in the recruitment of monocytes to the kidney. These findings suggest that PI3Kδ is a potential therapeutic target in AAV.

## INTRODUCTION

Antineutrophil cytoplasmic antibody–associated vasculitis (AAV) is a systemic disease affecting the joints, lungs, kidneys, skin, and other tissues and occurs most often in older adults. It is characterized by autoantibodies against the neutrophil and monocyte expressed antigens myeloperoxidase (MPO) and proteinase 3, which are thought to be pathogenic. The class I phosphoinositide 3‐kinase (PI3K) family mediate a wide range of cellular responses to cell surface receptors. The class IA PI3Ks are heterodimers composed of a catalytic subunit (p110α, p110β, or p110δ) combined with a p85 regulatory subunit. The class IB PI3Ks comprise the p110γ catalytic subunit combined with a p101 or p87/84 regulatory subunit. Mice deficient in p110γ were protected from disease in a mouse model of AAV, and a selective PI3Kγ inhibitor AS605240 also inhibited disease, with effects on neutrophil activation (1).

Activated PI3Kδ syndrome is caused by dominant mutations that increase activity of PI3Kδ. The clinical manifestations of respiratory tract infections, bronchiectasis, lymphadenopathy, and lymphoma are largely explained by activating effects on lymphocytes. Mice deficient in p110δ have impaired humoral immune responses (2), and there are direct effects on B and T cell receptor signaling (3). There are also effects on Th1, Th2, and Th17 T cell subsets (4). Catalytic subunit p110δ is important in autoimmunity, as evidenced by the reduction in autoantibodies to collagen with p110δ blockade (5). Furthermore, mice deficient in p110δ are protected from experimental allergic encephalomyelitis (6) and the development of a lupus‐like disease in Lyn^−/−^ mice (7).

Experiments using dual pharmacologic inhibition of p110γ and p110δ have been effective in preclinical models of rheumatoid arthritis and systemic lupus erythematosus (8). However, it is difficult to separate effects on autoimmunity from effects on downstream inflammation in these models. One study in a lupus nephritis model suggests specific effects of p110δ on renal monocyte/macrophage recruitment to the kidney (9). In the passive murine anti‐MPO model, disease is induced by injection of anti‐MPO antibody. In addition to providing confirmation of the pathogenicity of AAV, this model is ideal for the study of downstream inflammatory pathways. A limitation of the model is a lack of extrarenal manifestations. We have previously used this model to explore the role of granulocyte colony‐stimulating factor (G‐CSF) and complement (10, 11), but the role of p110δ has not been explored in the context of AAV. We therefore decided to study the effect of p110δ inactivation in the passive murine anti‐MPO model.

## MATERIALS AND METHODS

### Mice

**Figure 1:**
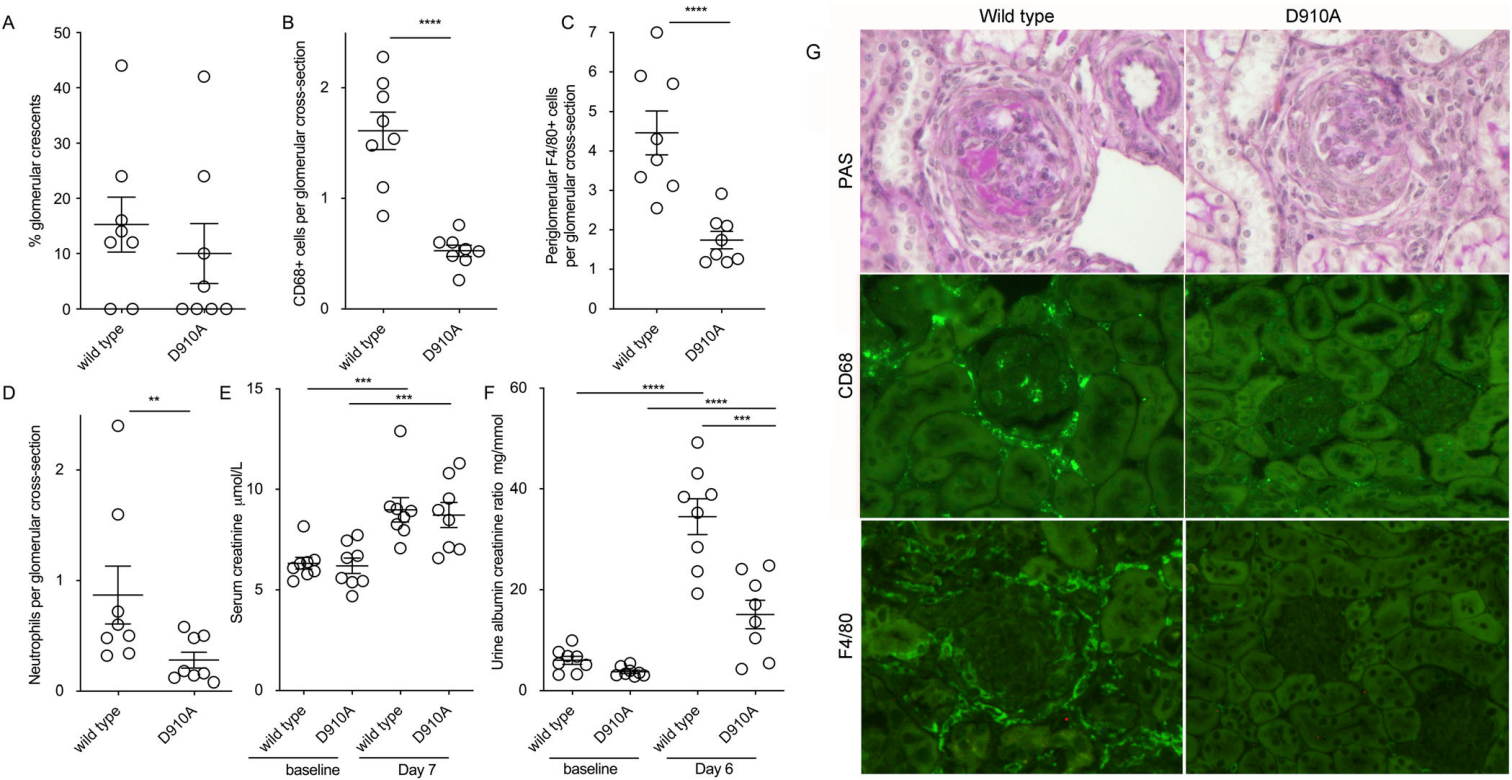
Antimyeloperoxidase (anti‐MPO) vasculitis induced by mouse anti‐MPO IgG in D910A and wild‐type mice. **A–D**, Histologic parameters assessed included glomerular crescents (**A**), glomerular CD68+ macrophages (**B**), periglomerular F4/80+ macrophages (**C**), and glomerular neutrophils (**D**). **E** and **F**, Biochemical parameters included serum creatinine on day 7 (**E**) and albuminuria on day 6 (**F**). **G**, Representative images show periodic acid–Schiff (PAS)–stained sections and immunofluorescence staining for CD68 and F4/80 in wild‐type and D910A mice with anti‐MPO vasculitis. Original magnification ×400. In **A–F**, symbols represent individual mice (n = 8 per group). Bars show the mean ± SEM. In **E** and **F**, a 2‐way analysis of variance with Šidák's multiple comparisons test was used to compare baseline data with day 6 or 7 data. Day 6 urine albumin:creatinine ratios and day 7 serum creatinine levels were compared using Student's *t*‐test, as were baseline values. ** = *P* < 0.01; *** = *P* < 0.001; **** = *P* < 0.0001.

D910A mice (2) backcrossed ≥10 generations to C57BL/6J and wild‐type C57BL/6J control mice were bred at the Babraham Institute in Cambridge and transferred to King's College London for the experiment featured in Figure 1 and Supplementary Figure 1 (available on the *Arthritis & Rheumatology* website at https://onlinelibrary.wiley.com/doi/10.1002/art.42298). For the other experiments, D910A mice were obtained from University College London and bred at King's College London, with C57BL/6J control mice from Charles River (Margate, UK). C57BL/6 mice congenic for CD45.1 (B6.SJL‐Ptprc^a^ Pepc^b^/BoyJ) were obtained from The Jackson Laboratory and bred in‐house. Female age‐matched mice ages 8–12 weeks were used in all experiments. Cages were located together for all experiments, but strain identities were not concealed. Experiments were approved by the local Animal Welfare and Ethics Review Board (with lay representation) and the UK Home Office (project license P4D019509).

### Induction of disease

**Figure 2:**
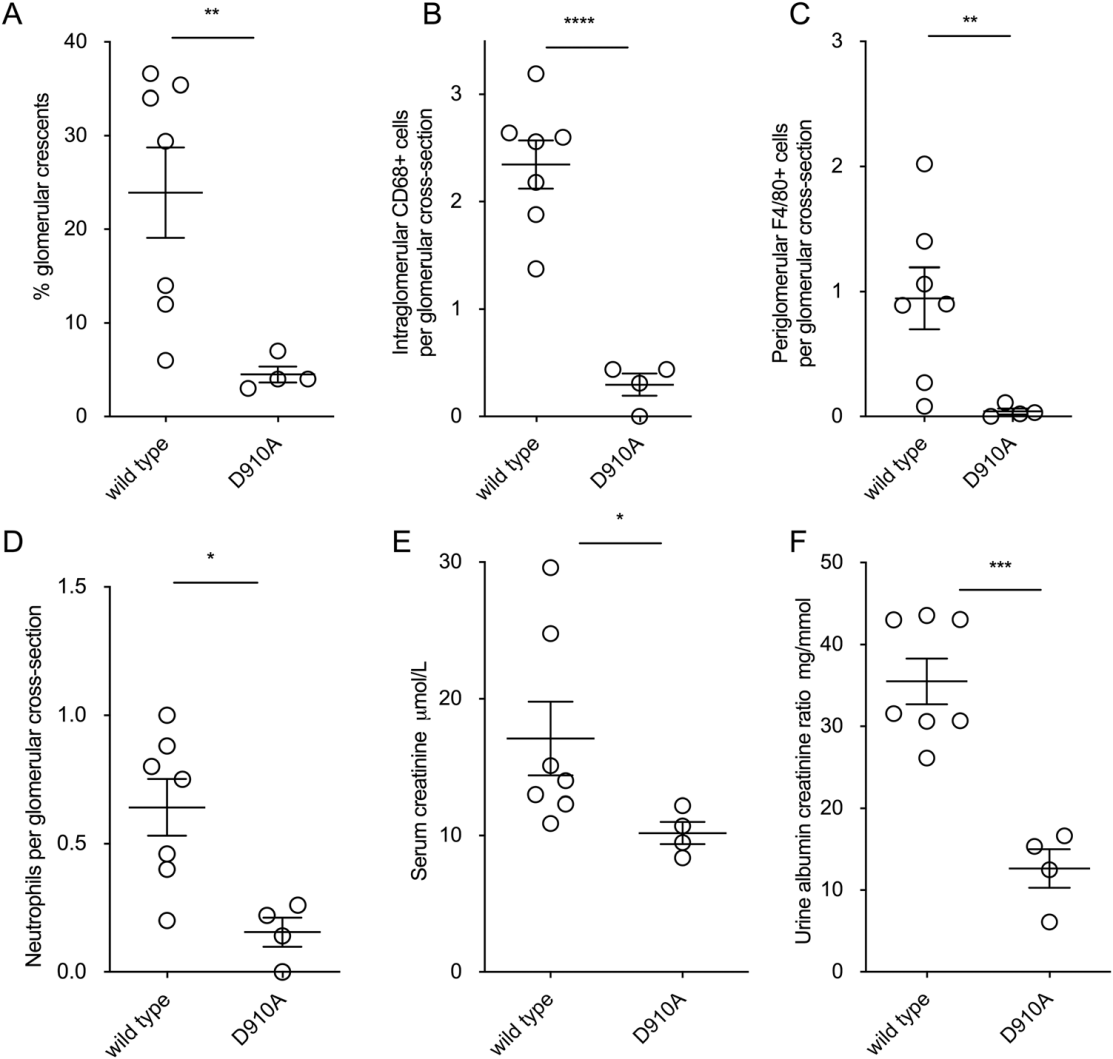
Antimyeloperoxidase (anti‐MPO) vasculitis induced by sheep anti‐MPO IgG in D910A and wild‐type mice. **A–D**, Histologic parameters assessed included glomerular crescents (**A**), glomerular CD68+ macrophages (**B**), periglomerular F4/80+ macrophages (**C**), and glomerular neutrophils (**D**). **E** and **F**, Biochemical parameters included serum creatinine on day 7 (**E**) and albuminuria on day 6 (**F**). Symbols represent individual mice (n = 7 total, and 4 per group). Bars show the mean ± SEM. * = *P* < 0.05; ** = *P* < 0.01; *** = *P* < 0.001; **** = *P* < 0.0001.

**Figure 3:**
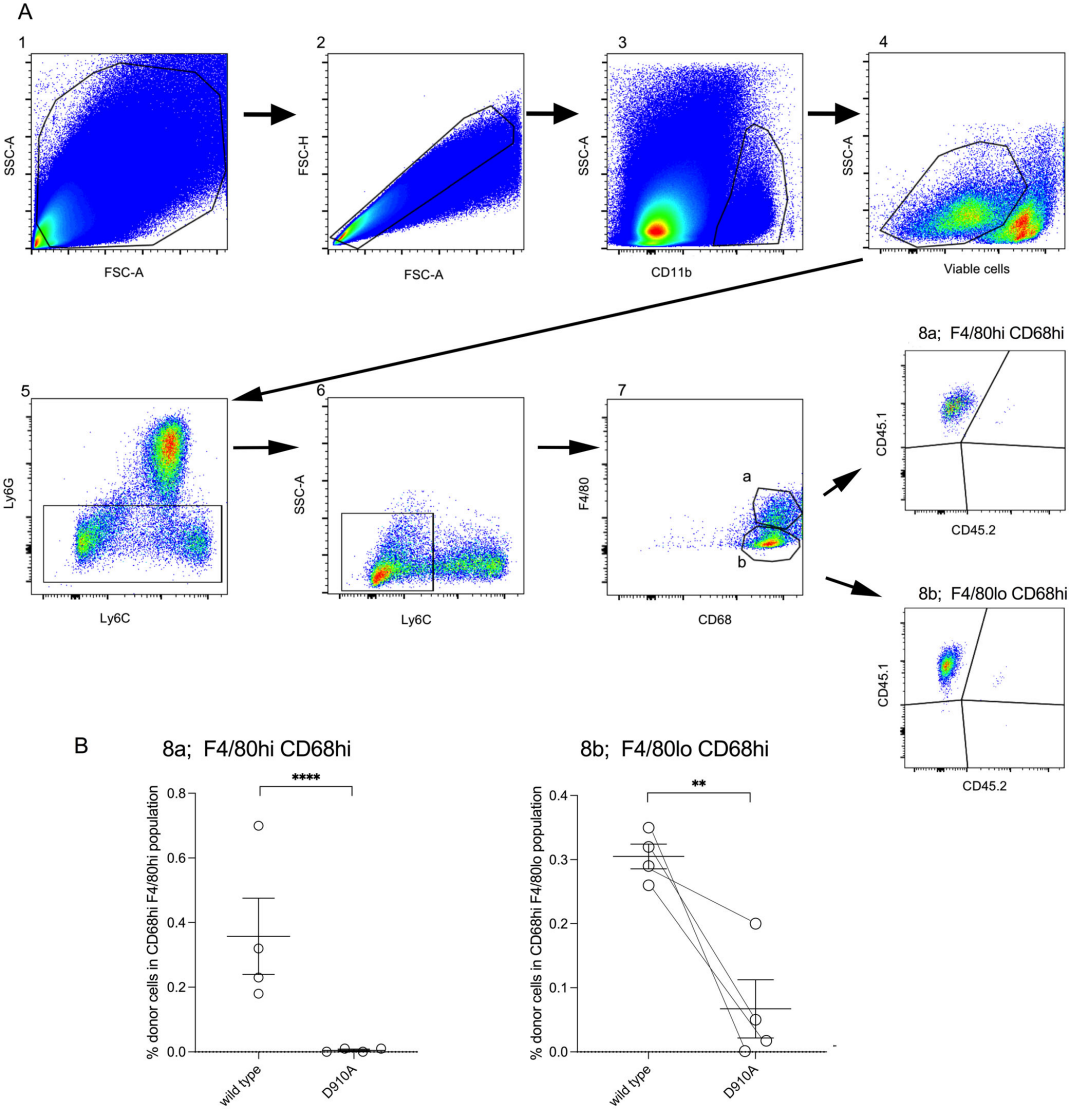
Recruitment of adoptively transferred bone marrow–derived monocytes from D910A and wild‐type mice in antimyeloperoxidase vasculitis. **A**, The gating strategy for flow cytometric analysis of digested kidneys taken on day 7 is shown, with gates numbered in order. Wild‐type recipient mice were CD45.2−CD45.1+, wild‐type donor mice were CD45.2+CD45.1+, and D910A donor mice were CD45.2+CD45.1−. Each recipient mouse received monocytes from both wild‐type and D910A donors. **B**, Data on CD68^high^ F4/80^high^ cells from gate 8a (left) and data on CD68^high^ F4/80^low^ cells from gate 8b (right) are shown, with lines joining symbols that represent data from the same animal (n = 4). Bars show the mean ± SEM. ** = *P* < 0.01; **** = *P* < 0.0001. Color figure can be viewed in the online issue, which is available at http://onlinelibrary.wiley.com/doi/10.1002/art.42298/abstract.

Anti‐MPO antibody was raised in MPO‐deficient mice as described (10), and IgG was purified by protein G chromatography (HiTrap; GE Healthcare). In the experiment shown in Figure 1, pegylated G‐CSF 30 μg (Neulasta; Amgen) was administered subcutaneously 8 and 4 days prior to disease induction, on the day of induction, and 4 days after induction. On day 0 (the day of disease induction), 2 mg/20 gm anti‐MPO IgG raised in mice was given intravenously, and 10 μg lipopolysaccharide (*Escherichia coli* R515, ALX‐581‐007‐L002; Enzo Life Sciences) was given intraperitoneally. Blood was taken from the saphenous vein on day −1. For the experiment in Figure 2, sheep were immunized with murine MPO (purified as previously described [10]) by IG Innovations (Ceredigion, Wales, UK). Mice were injected intravenously with 200 μl/20 gm of heat inactivated serum on day 0. Mice were treated with 6 μg G‐CSF (Neupogen; Amgen) subcutaneously daily from days −1 to 6. Lipopolysaccharide (*E coli* R515) was administered intraperitoneally at 10 μg/20 gm on days 0 and 3. Mice were euthanized on day 7. For the adoptive transfer experiments (Figure 3), mice were treated with 6 μg G‐CSF subcutaneously daily from days −4 to 6. On day 0, 2 mg/20 gm anti‐MPO IgG raised in mice was given. Lipopolysaccharide was given intraperitoneally at 10 μg/20 gm on days 0 and 3.

### Assessment of disease

Spot urines for the albumin:creatinine ratio were obtained on day 6, and mice were euthanized on day 7, with blood collected from the axillary vessels under terminal anesthesia. Kidneys were fixed in Bouin's solution, and paraffin‐embedded sections were stained with periodic acid–Schiff (PAS). A minimum of 100 glomeruli per animal were assessed to score the percentage of glomerular crescents and 50 glomeruli to obtain the number of neutrophils. Neutrophils were identified by their characteristic morphology on PAS sections. Periodate–lysine–paraformaldehyde–fixed tissue was used for immunofluorescence, with a minimum of 20 glomeruli per sample assessed in each case. Unlabeled primary antibodies were CD68 (clone FA11; Serotec), F4/80 (clone BM8; eBioscience), and C3 (clone RmC11H9; Cedar Lane). Detection was conducted with DyLight 488–conjugated mouse anti‐rat IgG (Jackson ImmunoResearch). Direct immunofluorescence staining was performed with fluorescein isothiocyanate (FITC)–conjugated goat anti‐mouse IgG (Fc‐specific) and FITC‐conjugated donkey anti‐sheep IgG (both from Jackson ImmunoResearch). All slides were coded before scoring so the researcher performing the histologic assessment was unaware of sample identity.

Serum creatinine was measured using liquid chromatography with tandem mass spectrometry (Pediatric Clinical Chemistry Laboratory at Guy's and St. Thomas’ NHS Foundation Trust), as was urine creatinine, for the data shown in Figure 2. For the data shown in Figure 1, urine creatinine was measured with a commercial creatinine assay using a plate reader and methodology based on instructions of the manufacturer (Diazyme), with a standard curve generated for all assays. Urine albumin was measured by enzyme‐linked immunosorbent assay (ELISA) (Bethyl Laboratories). Monocyte chemotactic protein 1 (MCP‐1) and CD163 were measured by ELISA (DuoSet) according to instructions of the manufacturer (Bio‐Techne). For details on circulating leukocytes, please refer to the Supplementary Methods (available on the *Arthritis & Rheumatology* website at https://onlinelibrary.wiley.com/doi/10.1002/art.42298).

### Adoptive transfer experiments

Hind limbs were collected, bone marrow cells were extracted, and monocytes were isolated using a CD115 MicroBead kit (Miltenyi Biotec). Recipient mice were injected intravenously with 1 × 10^7^ CD115+ cells from each of the donor strains on day 1 after disease induction. Mice were euthanized on day 7, and kidneys were collected for histology and kidney digestion.

### Kidney digestion and flow cytometry

Kidney digestion is described in the Supplementary Methods (https://onlinelibrary.wiley.com/doi/10.1002/art.42298). Ten million cells from the kidney single‐cell preparations were incubated with Fixable Viability Dye eFluor780 (ThermoFisher). Cells were blocked with 1 μg/ml Fc receptor block (anti‐CD16/32 antibody; BD Biosciences) and stained with the following fluorochrome‐conjugated antibodies for surface markers: CD11b (clone M1/70; BioLegend), CD45.1 (clone A20; Biolegend), CD45.2 (clone I04; ThermoFisher), Ly6G (clone IA8; BioLegend), Ly6C (AL‐21; BD Biosciences), and F4/80 (clone BM8; eBioscience). Cells were permeabilized using a CytoFix/CytoPerm kit (BD Biosciences), blocked with anti‐CD16/32 antibody (BD Biosciences), and incubated with anti‐CD68 (clone FA/11; BD Biosciences). Samples were run on an LSRFortessa using FACSDiva software (BD Biosciences), and data were analyzed using FlowJo software.

### Statistical analysis

GraphPad Prism version 9 was used for analyses. Student's unpaired *t*‐test was used to compare 2 groups, unless another test is specified. Some data were logarithmically transformed before analysis.

## RESULTS

### Protection from anti‐MPO vasculitis in D910A mice with mild disease

Anti‐MPO vasculitis was induced in mice with inactive p110δ (D910A) and in wild‐type mice. Immunofluorescence staining included both F4/80, a mature murine macrophage marker found in a predominantly periglomerular location (12), and CD68, which is found on all glomerular macrophages. Although there was no difference in glomerular crescents (Figure 1A), there were fewer glomerular CD68+ cells (Figure 1B), fewer periglomerular F4/80+ cells (Figure 1C), and fewer neutrophils (Figure 1D) in D910A mice. Serum creatinine was increased in both groups compared with baseline, but there was no difference between groups (Figure 1E). However, there was significantly less albuminuria in D910A mice compared with wild‐type controls (Figure 1F). Figure 1G shows representative histologic sections and immunofluorescence staining for CD68 and F4/80.

### No differences in circulating leukocytes

Peripheral blood leukocytes in D910A mice and wild‐type mice were compared. There were no differences in total white cells, peripheral total blood monocytes, monocyte subsets, or neutrophils in untreated mice (Supplementary Figures 1A–E, https://onlinelibrary.wiley.com/doi/10.1002/art.42298). We also assessed neutrophil numbers on day –1, before disease induction and after G‐CSF treatment for the experiment shown in Figure 1. No differences between wild‐type and D910A mice were seen (Supplementary Figure 1F). These results show that the differences in glomerular macrophages and neutrophils did not simply reflect differences in circulating leukocytes.

### Protection from acute kidney injury in D910A mice with more severe disease

Anti‐MPO vasculitis was induced with sheep anti‐MPO IgG to induce more severe disease, because we had not shown protection from acute kidney injury in our initial experiment. Using sheep IgG allowed for the injection of more IgG and led to more severe disease in wild‐type mice. The use of sheep anti‐MPO antibody did not result in glomerular deposition of sheep IgG (Supplementary Figure 2, https://onlinelibrary.wiley.com/doi/10.1002/art.42298). Weak staining for mouse IgG and C3 was seen in a mesangial pattern in mice with disease due to either mouse or sheep anti‐MPO IgG, along with tubular C3 (Supplementary Figure 2). This pattern of IgG and C3 staining is also seen in untreated mice. Therefore, this remained a good model of anti‐MPO vasculitis. Disease was less severe in D910A mice according to all experimental read outs. There were fewer glomerular crescents, fewer glomerular and periglomerular macrophages, fewer neutrophils, lower serum creatinine levels, and lower levels of albuminuria in D910A mice compared with wild‐type mice (Figure 2). These results confirmed that D910A mice were protected and that this included protection from glomerular crescent formation and acute kidney injury.

### Deficient recruitment of adoptively transferred bone marrow–derived monocytes from D910A mice in anti‐MPO vasculitis

The most striking finding in the first experiment, with disease induced by murine anti‐MPO IgG, was the lack of CD68+ and F4/80+ macrophages in and around glomeruli in D910A mice. This was observed despite the moderate disease severity. In view of their role in monocyte function, we measured MCP‐1 and CD163 in serum from the experiment shown in Figure 2, and there were no significant differences (Supplementary Figure 3, https://onlinelibrary.wiley.com/doi/10.1002/art.42298). MCP‐1 and CD163 were both undetectable in urine. Despite this, we decided to directly examine the recruitment of inflammatory monocytes in an adoptive transfer experiment. Each recipient mouse received bone marrow–derived monocytes from both wild‐type and D910A donors on day 1 after disease induction and was euthanized on day 7.

This experimental design meant that in each recipient mouse with anti‐MPO vasculitis, we could assess the relative recruitment to the kidney of adoptively transferred wild‐type and D910A monocytes. Paired donor‐derived cells from both strains experienced the same inflammatory environment and were extracted from the same kidney with a given disease severity. Significant disease was induced in all 4 mice with 9%, 20%, 28%, and 22% of glomerular cross sections having crescents on day 7, respectively. Figure 3A shows the gating strategy for flow cytometric analysis of digested kidneys taken on day 7. We identified populations of CD68^high^ F4/80^low^ and CD68^high^ F4/80^high^ cells corresponding to glomerular and periglomerular macrophages seen on immunofluorescence staining of tissue (Figure 1G). The data in Figure 3B show that there were significantly fewer CD68^high^ F4/80^low^ and CD68^high^ F4/80^high^ cells derived from transferred D910A monocytes than from wild‐type monocytes. These data confirm that monocytes with an inactive p110δ have a reduced recruitment to the inflamed kidney in anti‐MPO vasculitis.

## DISCUSSION

We have demonstrated that mice with an inactive p110δ are protected from vasculitis induced by passive transfer of anti‐MPO antibody. There was a striking reduction in renal macrophages and adoptive transfer experiments showed a defect in the recruitment of monocytes with an inactive p110δ to the kidney. Our results are consistent with previous data in a lupus model (9). In this study, there was a modest reduction in autoantibody levels with p110δ inhibition but a striking reduction in renal macrophages. The current data and findings from this previous study both suggest that p110δ is required for downstream inflammation in glomerulonephritis. In the previous report, in vitro data also suggested that p110δ was required for macrophage transmigration.

Previous in vitro data on human cells have shown a requirement for p110δ in monocyte adhesion and transmigration (13). Pharmacologic inhibition of p110δ decreased migration of both primary peripheral blood derived monocytes and THP‐1 cells. Further experiments with THP‐1 cells and a coronary artery endothelial cell layer showed that, in the absence of chemokines, PI3Kδ regulates the activation of β1 integrins mediating the binding of monocytes to vascular cell adhesion molecule 1, while in the presence of MCP‐1, PI3Kδ regulates β2 integrin activation and adhesion to intracellular adhesion molecule 1. We have previously shown that anti‐MPO IgG from patients affect macrophage development (14), and p110δ could play a role in mediating the response of monocytes to anti‐MPO IgG. However, the previous in vitro data in both murine and human cells discussed above (9, 13) suggest a more general effect on monocyte recruitment which could be relevant to other diseases. We have not identified the molecular mechanism by which p110δ enhances monocyte recruitment. An understanding of this will require further work beyond this brief report.

We focused on monocytes, and the most striking finding was a reduction in CD68+ and F4/80+ cells in the kidney in our initial experiments. However, we have not excluded a role for p110δ in neutrophils in addition to monocytes. A previous study using the murine anti‐MPO model suggested that neutrophil depletion was protective (15). However, the depleting antibody used (NIMP‐R14) would have also affected monocytes, so the relative importance of monocytes and neutrophils was not clear. We have shown that neutrophils are essential in the autologous nephrotoxic nephritis model of crescentic glomerulonephritis using mice with a genetic deficiency of neutrophils (16). Therefore, it seems likely that neutrophils are required in anti‐MPO vasculitis.

The current study does not explore the effect of p110δ deficiency on autoimmunity to MPO, and one would predict that it will inhibit this in addition to the antiinflammatory effects we have demonstrated. PI3Kδ inhibitors are being studied with idelalisib (CAL‐101), an older PI3Kδ inhibitor, which is approved for hematologic malignancy. However, adverse effects limit its potential as a therapy for AAV. Several PI3Kδ inhibitors are available that do not appear to have these side effects and could inhibit both autoimmunity and inflammation in AAV.

Therapies that target p110δ and p110γ are being developed for hematologic malignancies in addition to autoimmune disease. This is an advantage since safety data will accumulate at a faster rate because of this diverse clinical application. The major unmet need in AAV is for antiinflammatory therapies to reduce or eliminate the need for glucocorticoids. There has been progress in this respect, with positive results for the C5a receptor antagonist avacopan. However, additional treatments that target other pathways will be useful. The current study and previous work (1) suggest that p110δ and p110γ are both potential therapeutic targets. They may be more effective and serve as an alternative if avacopan is not tolerated or provide additional benefit when used in combination.

## AUTHOR CONTRIBUTIONS

All authors were involved in drafting the article or revising it critically for important intellectual content, and all authors approved the final version to be published. Dr. Robson had full access to all of the data in the study and takes responsibility for the integrity of the data and the accuracy of the data analysis.

### Study conception and design

Robson.

### Acquisition of data

Flórez‐Barrós, Freeley, Tham.

### Analysis and interpretation of data

Flórez‐Barrós, Freeley, Tham, Robson.

## Supporting information


Disclosure Form

**Appendix S1** Supplementary Information

## References

[1] Schreiber A , Rolle S , Peripelittchenko L , et al. Phosphoinositol 3‐kinase‐γ mediates antineutrophil cytoplasmic autoantibody‐induced glomerulonephritis. Kidney Int 2009;77:118–28. 19907415 10.1038/ki.2009.420

[2] Okkenhaug K , Bilancio A , Farjot G , et al. Impaired B and T cell antigen receptor signaling in p110δ PI 3‐kinase mutant mice. Science 2002;297:1031–4. 12130661 10.1126/science.1073560

[3] Ramadani F , Bolland DJ , Garcon F , et al. The PI3K isoforms p110α and p110δ are essential for pre‐B cell receptor signaling and B cell development. Sci Signal 2012;3:ra60. 10.1126/scisignal.2001104 PMC3540743 20699475

[4] Soond, DR , Bjorgo E , Moltu K , et al. PI3K p110δ regulates T‐cell cytokine production during primary and secondary immune responses in mice and humans. Blood 2010;115:2203–13. 20081091 10.1182/blood-2009-07-232330 PMC3593196

[5] Durand CA , Hartvigsen K , Fogelstrand L , et al. Phosphoinositide 3‐kinase p110 δ regulates natural antibody production, marginal zone and B‐1 B cell function, and autoantibody responses. J Immunol 2009;183:5673–84. 19843950 10.4049/jimmunol.0900432

[6] Haylock‐Jacobs S , Comerford I , Bunting M , et al. PI3Kδ drives the pathogenesis of experimental autoimmune encephalomyelitis by inhibiting effector T cell apoptosis and promoting Th17 differentiation. J Autoimmun 2011;36:278–87. 21396797 10.1016/j.jaut.2011.02.006

[7] Maxwell MJ , Tsantikos E , Kong AM , et al. Attenuation of phosphoinositide 3‐kinase δ signaling restrains autoimmune disease. J Autoimmun 2012;38:381–91 22537464 10.1016/j.jaut.2012.04.001

[8] Stark AK , Sriskantharajah S , Hessel EM , et al. PI3K inhibitors in inflammation, autoimmunity and cancer. Curr Opin Pharmacol 2015;23:82–91. 26093105 10.1016/j.coph.2015.05.017 PMC4518027

[9] Suarez‐Fueyo A , Rojas JM , Cariaga AE , et al. Inhibition of PI3Kδ reduces kidney infiltration by macrophages and ameliorates systemic lupus in the mouse. J Immunol 2014;193:544–54 24935930 10.4049/jimmunol.1400350

[10] Freeley SJ , Coughlan AM , Popat RJ , et al. Granulocyte colony stimulating factor exacerbates antineutrophil cytoplasmic antibody vasculitis. Ann Rheum Dis 2013;72:1053–8. 23087180 10.1136/annrheumdis-2012-202160

[11] Freeley SJ , Popat RJ , Parmar K , et al. Experimentally‐induced anti‐myeloperoxidase vasculitis does not require properdin, MASP‐2 or bone marrow‐derived C5. J Pathol 2016;240:61–71. 27235854 10.1002/path.4754 PMC4996338

[12] Masaki T , Chow F , Nikolic‐Paterson DJ , et al. Heterogeneity of antigen expression explains controversy over glomerular macrophage accumulation in mouse glomerulonephritis. Nephrol Dial Transplant 2003;18:178–81. 12480978 10.1093/ndt/18.1.178

[13] Ferreira AM , Isaacs H , Hayflick JS , et al. The p110δ isoform of PI3K differentially regulates β1 and β2 integrin‐mediated monocyte adhesion and spreading and modulates diapedesis. Microcirculation 2006;13:439–56. 16864411 10.1080/10739680600776062

[14] Popat RJ , Hakki S , Thakker A , et al. Anti‐myeloperoxidase antibodies attenuate the monocyte response to LPS and shape macrophage development. JCI Insight 2017;2:e87379. 28138552 10.1172/jci.insight.87379 PMC5256146

[15] Xiao H , Heeringa P , Liu Z , et al. The role of neutrophils in the induction of glomerulonephritis by anti‐myeloperoxidase antibodies. Am J Pathol 2005;167:39–45. 15972950 10.1016/S0002-9440(10)62951-3 PMC1603451

[16] Tham EL , Freeley SJ , Bearder S , et al. VISTA deficiency protects from immune complex‐mediated glomerulonephritis by inhibiting neutrophil activation. J Autoimmun 2020;113:102501. 32586651 10.1016/j.jaut.2020.102501

