# Supplementary material for: Phosphatidylinositol 3‐Kinase δ Deficiency Protects From Antimyeloperoxidase Vasculitis
Source: Arthritis Rheumatol. 2022 Nov 18;75(1):64–70. doi: 10.1002/art.42298 (PMC10099887; doi:10.1002/art.42298)
Supplement: Supplementary file 2 — Appendix S1 Supplementary Information [file ART-75-64-s002.docx]

**Supplementary Methods and Data**

*Circulating leukocytes*

For assessing circulating leukocytes blood samples were taken from the saphenous vein into EDTA-coated tubes. Total white cell counts were determined using a haemocytometer and EDTA anticoagulated blood diluted in Turk's solution. Whole blood was stained with the following conjugated antibodies: CD11b (clone M1/70, eBioscience), Ly6G (clone 1A8, BD Biosciences), Ly6C (clone AL-21 BD Bioscience). Red cells were lysed using FACS Lysing Solution (Becton Dickinson) according to the manufacturer's instructions. Neutrophils were identified as CD11b+Ly6G+ cells. Monocytes were identified as CD11b+L6G-Ly6C+ cells. They were further subdivided into Ly6C high and low subsets. Absolute numbers of neutrophils and monocytes were calculated from the total white cell count and the percentage of neutrophils or monocyte subsets. Samples were run on a LSR Fortessa using FacsDiva software (BD Biosciences) and data analyzed using FlowJo software (BD Life Sciences).

*Kidney Digestion*

Kidneys were removed and placed in cold FACS buffer. Kidneys were decapsulated and the cortex was trimmed and finely chopped with surgical scissors. They were added to 1 ml of preheated dissociation media (HBSS, Sigma Aldrich; 3mg/ml Collagenase/Dispase, Sigma; 0.2 mg/ml DNAse type I, Sigma; 50μM CaCl2, Sigma) and incubated at 37°C on MACSmix Tube Rotator (Miltenyi Biotec) for 30 minutes. Samples were subjected to mechanical digestion with surgical scissors before a further period of gentle shaking at 37°C for 20 minutes with extra fresh dissociation media. The tissue preparations were filtered through 70 μm and 40 μm strainer (BD Bioscience) to obtain single cell suspensions and centrifuged at 500g for 10 minutes. After red cell lysis, cells were resuspended in RPMI (Sigma) supplemented with 10% Fetal Bovine Serum (Sigma) and 2mM EDTA (Sigma).


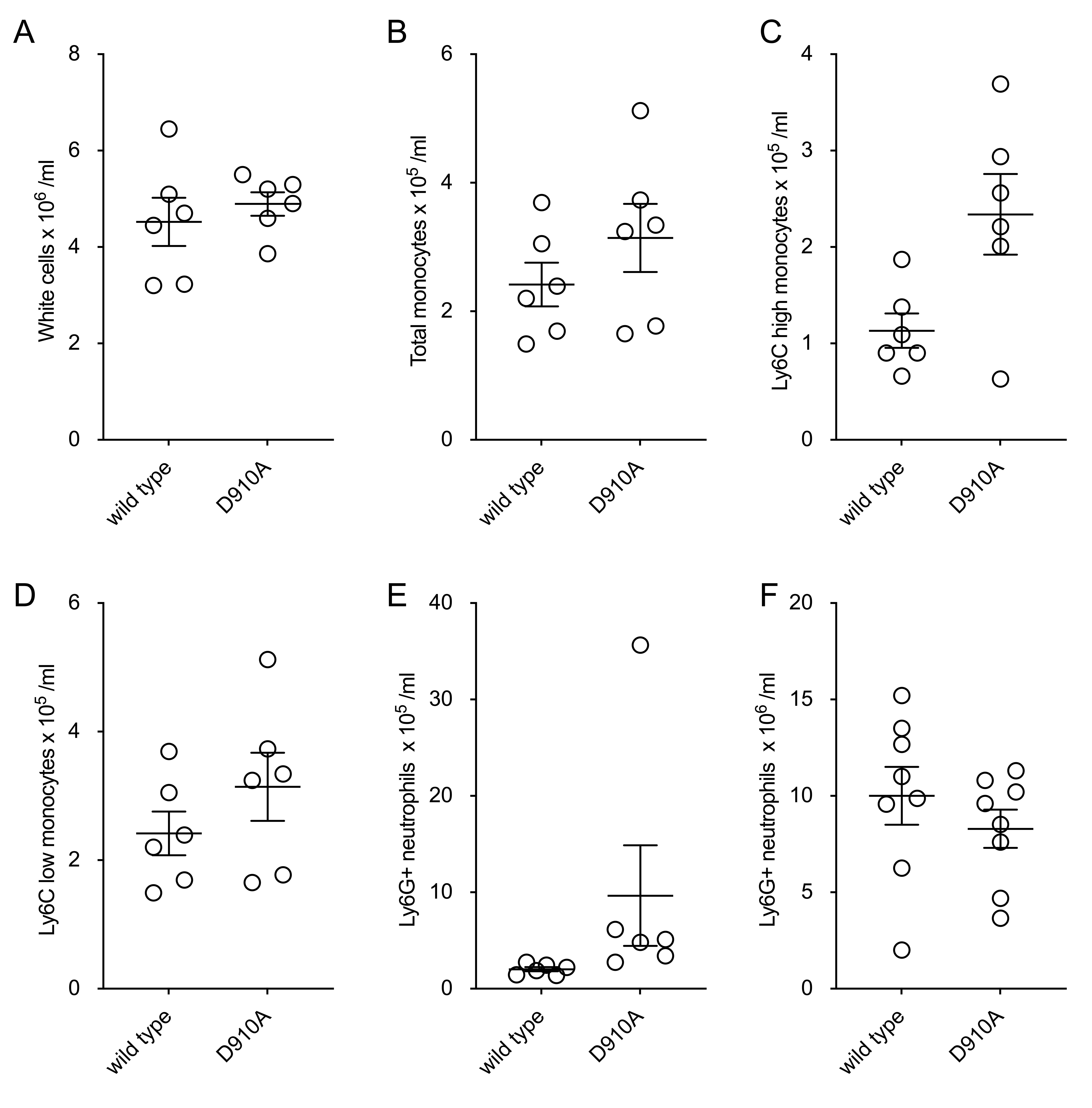


Figure S1. Circulating leukocytes in D910A mice compared with wild types. (A-E) Data from untreated mice. (A) Total leukocytes, (B) Total monocytes, (C) Ly6C high monocytes, (D) Ly6C low monocytes, (E) Neutrophils. (F) Neutrophils in peripheral blood taken after G-CSF administration and the day before disease induction for the experiment shown in Figure 1. Each symbol is an individual mouse. Error bars are Mean±SEM.


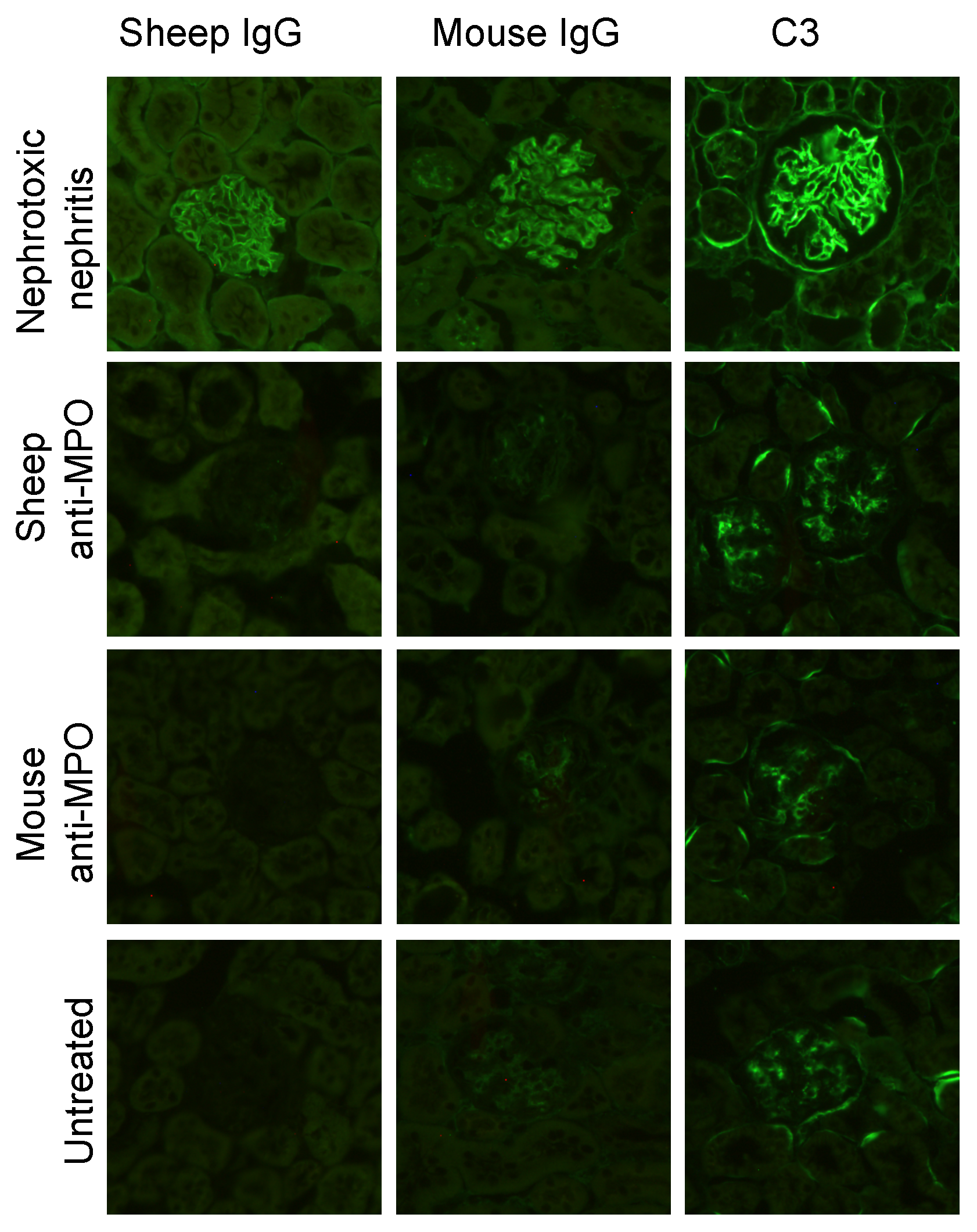


Figure S2. Immunofluorescence staining for sheep IgG, mouse IgG and C3 in mice with anti-MPO vasculitis induced by mouse or sheep anti-MPO IgG (day 7), along with untreated mice. Sections from three mice in each group were stained, with similar findings in each. Sections from mice with nephrotoxic nephritis are included as a positive control.


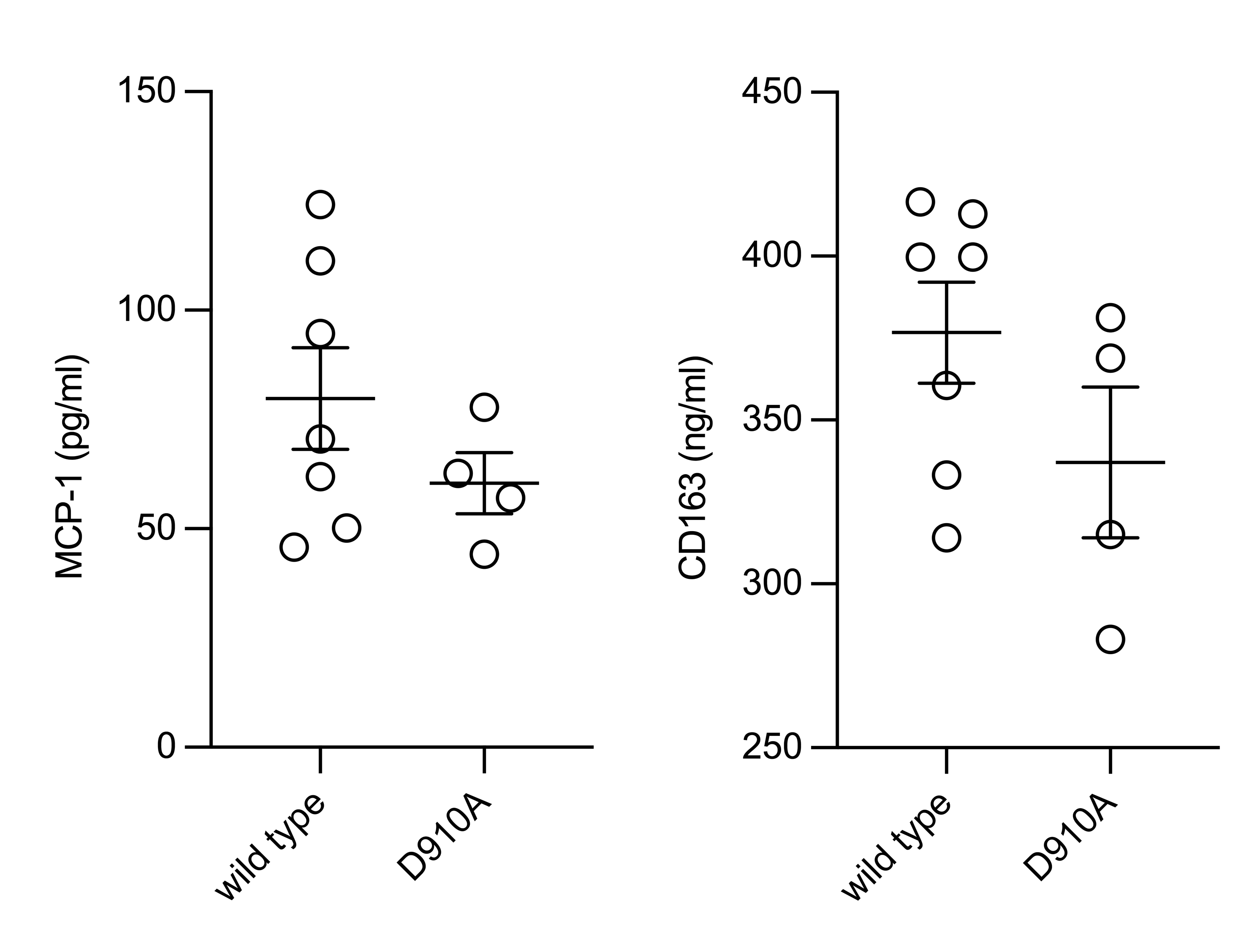


Figure S3. Serum levels of MCP-1 and CD163 at day 7 from mice in the experiment shown in Figure 2. Each symbol is an individual mouse. Error bars are Mean±SEM.
